# Gene Module Identification from Microarray Data Using Nonnegative Independent Component Analysis

**Published:** 2008-01-15

**Authors:** Ting Gong, Jianhua Xuan, Chen Wang, Huai Li, Eric Hoffman, Robert Clarke, Yue Wang

**Affiliations:** 1 Department of Electrical and Computer Engineering, Virginia Polytechnic Institute and State University, Arlington, VA 22203, U.S.A; 2 Bioinformatics Unit, Research Resources Branch, National Institute on Aging, NIH, Baltimore, MD 21224, U.S.A; 3 Research Center for Genetic Medicine, Children’s National Medical Center, Washington, DC 20010, U.S.A; 4 Department of Oncology and Physiology and Biophysics, Georgetown University School of Medicine, Washington, DC 20057, U.S.A

**Keywords:** transcriptional module, gene module identification, non-negative independent component analysis (nICA), visual statistical data analyzer (VISDA), muscle regeneration, microarray data analysisp

## Abstract

Genes mostly interact with each other to form transcriptional modules for performing single or multiple functions. It is important to unravel such transcriptional modules and to determine how disturbances in them may lead to disease. Here, we propose a non-negative independent component analysis (nICA) approach for transcriptional module discovery. nICA method utilizes the non-negativity constraint to enforce the independence of biological processes within the participated genes. In such, nICA decomposes the observed gene expression into positive independent components, which fits better to the reality of corresponding putative biological processes. In conjunction with nICA modeling, visual statistical data analyzer (VISDA) is applied to group genes into modules in latent variable space. We demonstrate the usefulness of the approach through the identification of composite modules from yeast data and the discovery of pathway modules in muscle regeneration.

## Introduction

Genes often interact with each other to carry out cellular activities (Segal et al. 2004). The set of genes tightly regulated in a specific cellular process can be considered as a process-specific transcriptional module (Hughes et al. 2000; Brunet et al. 2004; Segal et al. 2004). It is important to identify such modules for understanding biological events associated with different experimental conditions, which may further help identify gene expression signatures associated with diseases.

Various methods have been proposed to identify gene transcriptional modules from microarray data. Several clustering techniques, such as hierarchical clustering (Spellman et al. 1998), k-means (Saeed Tavazoie et al. 1999) and self-organizing maps (Tamayo et al. 1999), are in common use for identifying meaningful subgroup genes exhibiting similar expression patterns. These approaches played a key role in gaining insights into the biological mechanisms associated with different physiological states. However, the clustering approaches are not well tuned for regulatory module identification due to that: (1) a set of co-regulated genes may only co-express in a subset of experimental conditions, and (2) clustering the genes into one and only one group may also mask the interrelationships between genes that are assigned to different clusters but show local similarities in their expression patterns. Thus, biologists are more interested in finding the hidden regulatory patterns behind gene expression patterns, which strengthens the biological relevance of the grouped genes, i.e. the genes are co-regulated to form transcriptional modules.

Recently, matrix decomposition methods have been introduced to uncover transcriptional modules from microarray data, including independent component analysis (ICA) (Liebermeister, 2002; Lee and Batzoglou, 2003; Frigyesi et al. 2006) and nonnegative matrix factorizations (NMF) (Carmona-Saez et al. 2006; Lee and Seung, 1999; Kim and Tidor, 2003; Wang et al. 2006). These methods treat microarray data as a mixture of unknown factors (or components) that may correspond to specific biological processes. Specifically, the level of any given mRNA expression is modeled as the net sum of a complex superposition of cooperating and/or counteracting biological processes. ICA is a statistical method for revealing independent hidden factors that underlie sets of random variables or observations. In the context of microarray data, these statistically independent hidden factors may correspond to putative biological processes or transcriptional modules. It has been shown that the clusters found by ICA are directly associated with biological processes with common regulatory mechanisms (Lee and Batzoglou, 2003; Frigyesi et al. 2006). However, it is problematic to directly apply ICA to gene expression data due to its strong assumption of the independence of hidden variables in whole gene population. Biologically, it is more plausible to assume that the independence holds only for those genes that actively participating in biological processes. Therefore, we need to make further assumptions to constrain the ICA model for gene module identification.

In this paper, we propose to use non-negative ICA (nICA) for gene module identification (Ting et al. 2006; Oja and Plumbley, 2004; Plumbley, 2001). nICA exploits the non-negativity constraint to enforce the independence of biological processes within the participated genes. In principle, nICA can be thought as a projection method with which the expression levels (or ratios) are projected onto some new non-negative components with least statistical dependence. We believe that nICA provides a better model of gene expression data than ICA does, hence, more appealing for gene module identification.

In the proposed approach, we first perform input sample selection to improve the quality of separation of the components. We then develop a stability analysis procedure to determine the number of non-negative independent components. We further implement a learning algorithm of nICA with the non-negativity constraint for hidden component estimation. Finally, we use visual statistical data analyzer (VISDA), a data visualization and clustering tool (Wang et al. 2007b; Wang et al. 2003), to group the genes into modules in latent variable space. We demonstrate the effectiveness of the proposed approach for gene module identification using yeast and muscle regeneration datasets. The biological relevance of the identified gene modules is validated by functional annotation analysis. Compared with conventional ICA-based approach, the proposed approach appears to have improved performance in finding biologically meaningful transcriptional modules.

## Methods

**Figure 1:**
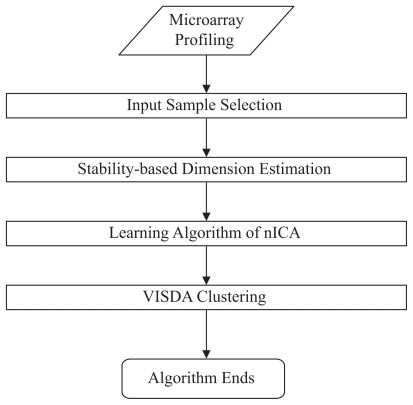
Flowchart of the proposed nICA approach for gene module identification.

The flowchart of the proposed approach is outlined in Figure 1. The algorithm consists of the following components - input sample selection, stability-based dimension estimation, learning algorithm of nICA, and gene clustering by VISDA. We provide a detailed description of each component as follows.

### Input sample selection

Input sample selection aims at selecting the most informative samples among the available samples for nICA decomposition. Without the proper selection of input samples, some computational problems such as increased computational complexity and degraded convergence may arise. Even worse, some samples may cause singularity problems for nICA decomposition. Principle components analysis (PCA), a variance based dimension reduction technique, is often used for input sample selection. But PCA is not always effective for nICA decomposition since the variance of a sample is not necessarily related to the importance of a sample.

Here, we propose to use mutual information (Vrins et al. 2003) to perform input sample selection. The objective is to select *m*′ informative samples (*v*_1_,…,*v_m_*,) from a set of m samples (*x*_1_,…, *x_m_*), where *m* > *m*′. At each step of the algorithm, we choose a sample that is as statistically independent as possible (Hyvärinen et al. 2001) from the already selected samples *v_j,_* *j* = 1,…, *k*−1. In other words, *x_i_* is the *k-th* selected sample (i.e. *v_k_*) if the following cost function *f* (*i*, *k*−1) (defined as the sum of mutual information) is minimized when *i* = *l*:

(1) $$\begin{array}{l} f\left(i,k-1\right)=\sum_{j=1}^{k-1}MI\left(x_{i},v_{j}\right)\\ \text{for all }x_{i}\notin \left\{v_{j},j=1,\ldots ,k-1\right\}, \end{array}$$

where *MI(.,.)* denotes the mutual information that is defined as (Cover and Thomas, 1991):

(2) $$MI\left(x_{i},v_{j}\right)=H\left(x_{i}\right)+H\left(v_{j}\right)-H\left(x_{i},v_{j}\right).$$

In Eq. (2), *H*(*x_i_*) (*H*(*v_i_*)) represents the entropy of a centered univariate random variable *x_i_* (*v_i_*) and *H*(*x_i_*,*v_j_*) represents the joint entropy of two centered univariate random variable *x_i_* and *v_j_* (Bach and Jordan, 2003). Therefore, the selected subset (*v*_1_,…,*v_m_,*) will contain the samples that are mutually “quite different” as a result of the minimization of mutual information.

### Stability-based dimension estimation

**Figure 2:**
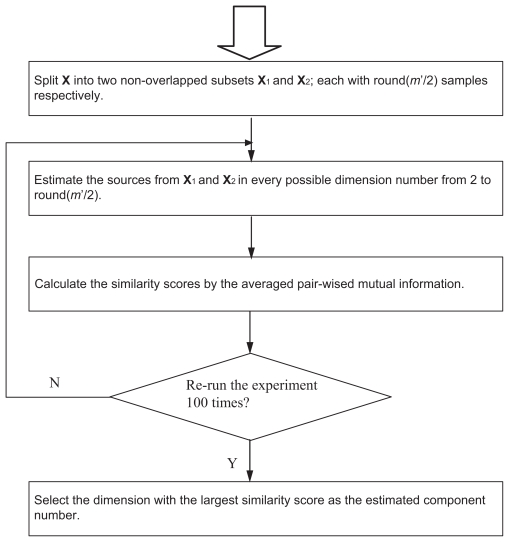
General schema of “splitting by samples” for dimension estimation.

In practice, the number of independent components for nICA is often determined by the user’s prior knowledge or obtained by PCA with a criterion of containing 95% of energy mainly to eliminate the noise effect (Hyvärinen et al. 2001). However, from our experience, it is often a difficult task in microarray data analysis to obtain a meaningful number of components by either the prior knowledge or PCA approach. When the number of components is incorrectly estimated, nICA will produce many possible false components for gene module identification. Hence, we propose to conduct stability analysis on gene expression data to estimate the number of components. Figure 2 shows the proposed stability-based schema, namely “splitting by samples”, for reliable dimension estimation of nICA (Wang et al. 2007a).

The basic idea of the stability-based approach is that the nICA results from two data subsets sampled from a common data set should be consistent. The consistency (or similarity) of the nICA results from two non-overlapped subsets reflects of the consistency between the assumed and underlying component numbers. More specifically, we split the samples into two non-overlapped subsets for nICA analysis and run the algorithm from *i* = 2 to full dimension of the subset of samples. We believe that if the dimension estimation really captures the underlying biological component number, the similarity score measured by mutual information between the components estimated from the two data subsets should give the best similarity score among all of the dimensions. When the estimated component number is not equal to the true number, the nICA results will show a tendency of mismatched components being estimated, hence, a decrease of similarity.

Due to the ambiguity of the scale in the nICA estimates, we need to normalize the estimated components and register them before calculating the similarity score. In our approach, we first normalize the estimated components to be unit-variance variables. We then perform the registration (or alignment) of two permutated versions of components via an information theoretic approach. The exact way to align (or register) different pairs of components is by examining their mutual information. We calculate the similarity score after the alignment using averaged pair-wise mutual information:

(3) $$Q=\frac{1}{\left\lfloor m^{\prime }/2\right\rfloor }\sum_{i=1}^{\left\lfloor m^{\prime }/2\right\rfloor }MI\left(s_{i}^{\left(1\right)},s_{i}^{\left(2\right)}\right),$$

where *MI(.,.)* denotes the mutual information estimate as defined in Eq. (2), ⌊· ⌋ is the floor function, and *s_i_*^(1)^, *s_i_*^(2)^, is the *i-th* aligned pair of the components estimated from two different subsets. In order to obtain a reliable estimation of the dimension number, stability tests are performed *P* times independently (in our experimental design, we re-run the algorithm *P* = 100 times with random initialization), each time after a random shuffling to the order of samples. Finally, we choose the dimension with the largest similarity score averaged over *P* runs as the estimate of the component number.

### Learning algorithm of nICA

We present a learning algorithm for nICA based on a latent model:

(4) X=AS,

where **X** denotes the microarray data matrix with rows corresponding to samples and columns corresponding to genes, **S** = (*s*_1_,…,*s_n_*)*^T^* represents the *n* independent biological processes, and **A** is the mixing matrix (matrix of contributions of each biological process). Suppose that *s_i_* (*i* = 1,…,*n*) has non-zero probability density function (pdf) all the way down to zero, then it has been proven (Oja and Plumbley, 2004; Plumbley, 2003; Plumbley, 2002) that we can find **Y** = **US** where **U** is a square orthonormal rotation and permutation matrix. It is equivalent to say that the elements **yi** of **Y** are a permutation of components if and only if all **yi** are all non-negative. The above result can be used to derive a simple solution to the nICA problem. Since **Y** = **US** can also be written as **Y** = **WZ** with **Z** the pre-whitened observation matrix and **W** an un-known orthogonal (rotation) de-mixing matrix, it suffices to find an orthogonal matrix **W** for which **Y** = **WZ** is non-negative. Therefore we can consider nICA as a procedure with the following two steps: 1) removing the second order statistics by whitening the data; 2) searching for a rotation matrix to make all the data fit into the positive quadrant.

A learning algorithm to find the de-mixing matrix **W** can be summarized as follows (Oja and Plumbley, 2004):

1. Pre-whiten the observed data **X** by the whitening matrix **V**:
  (5) Z=VX.
2. Define the cost function *J* as:
  (6) $$\begin{array}{l} J(W)=E\left\{{\left\|Z-W^{T}Y^{+}\right\|}^{2}\right\}\\ Y=\mathrm{WZ}\\ y_{i}^{+}=\text{max}\left(0,y_{i}\right)\\ Y^{+}=\left(Y_{1}^{+},Y_{2}^{+},\ldots ,Y_{\text{n}}^{+}\right), \end{array}$$
  and use a gradient descent algorithm to minimize the cost function *J* in Eq. (6):
  (7) $$\tilde{W}=W-\gamma \frac{\partial J}{\partial W},$$
  where *γ* is the step size.
3. Project the unconstraint gradient descent set onto a set of orthonormal vectors:
  (8) $$W={\left(\tilde{W}{\tilde{W}}^{T}\right)}^{-1/2}\tilde{W}.$$

### VISDA clustering

After performing nICA, we obtain the independent components representing some distinct biological processes. In these putative biological processes, the genes showing relatively high or low expression levels are most interesting. The analysis of gene patterns that are significantly over- or under-expressed in the components may provide insights into the biological events associated with these latent processes. We first use a pre-screening procedure to single out these genes and then apply VISDA to analyze the gene patterns in the latent space. In the pre-screening procedure, we first sort the genes by their contributions (or loads) in each component, which creates a natural ordination in which genes are arranged based on their association with a given component. Then we select a subset of genes within each component, i.e. the over-expressed genes or under-expressed genes according to the value of each gene in the component (Lee and Batzoglou, 2003). By taking the union of the selected genes from each component, we form a pool of genes that we believe are closely related to the biological processes revealed by nICA.

We then employ VISDA, a statistical model based clustering tool, to perform gene clustering on those selected genes in the latent space. Based on a hierarchical standard finite normal mixture (SFNM) model, VISDA captures the coherent structures in the latent space and performs top-down divisive clustering. The fitting process of the SFNM model is achieved by the Expectation Maximization (EM) algorithm (Wang et al. 2007b), which maximizes the likelihood function. For each cluster at a level of the hierarchy, VISDA uses two different projection methods, principle component analysis (PCA) and PCA-projection pursuit (PCA-PPM) (Wang et al. 2007b), to visualize the sub-clusters within the clusters. The user chooses one of the projections that he/she thinks better revealing the data structures. On the chosen projection, user initializes models with different number of clusters by clicking on the computer screen at the centers of the clusters. These two-dimensional (2-D) models will be refined by the EM algorithm and compete according to Minimum Description Length (MDL) criterion or human justification. The winning model in 2-D space will be transferred back to original data space to initialize the data model in that space. Then the EM algorithm in original data space will refine the model and obtain the partition of data at that level. At the top level, the whole dataset is split into several coarse clusters that may contain multiple functional modules; at lower levels, these coarse clusters are further decomposed into finer clusters, until no substructures can be found.

## Results

We applied the proposed nICA approach to identify gene functional modules from the following three expression datasets: Dataset 1 - budding yeast during cell cycle CLB_2_/CLN_3_ overactive stain (Spellman et al. 1998), consisting of spotted array measurements of 6,178 genes in 77 time points; Dataset 2 - yeast in various stressful conditions consisting of spotted array measurements of 6,152 genes in 173 experiments (Gasch et al. 2000); Dataset 3 - a 27-time points muscle regeneration series *in vivo* murine regeneration with Affymetrix oligonucleotide array measurements of 7,570 genes (Zhao et al. 2003). To determine whether the proposed approach can uncover the gene modules from gene expression data in the latent space, we mainly used the Biological Network Gene Ontology (BiNGO) tool (Maere et al. 2005) to evaluate the enrichment of functional annotations, and the Ingenuity Pathway Analysis (IPA) to assess the regulatory networks associated with the gene sets obtained by nICA.

### Yeast cell cycle data

The yeast cell-cycle dataset was preprocessed to obtain log-ratios between red and green intensities, i.e. *x_ij_* = log_2_(*r_ij_*/*g_ij_*). Since the data set contains both positive and negative log-ratio values, we need to perform data pre-treatment for nICA. We assume that distinct regulatory interactions are responsible for up-regulation versus down-regulation of gene expression. With the spirit of “divide and conquer”, we split the data into two parts - positive and negative values corresponding to up- and down-regulated gene sets respectively - to fit the nICA model.

**Figure 3:**
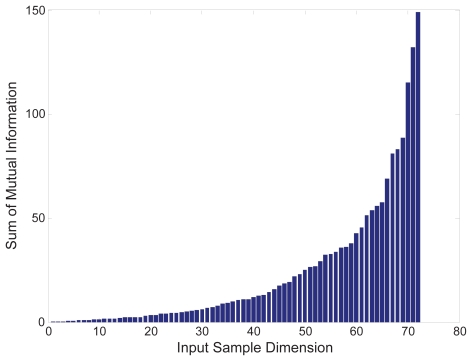
Input sample selection for the samples in the positive part of the cell cycle data set.

**Figure 4:**
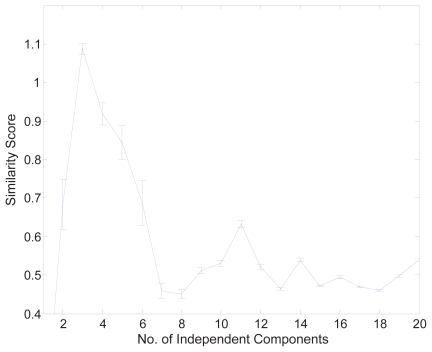
Stability analysis of the positive part of the cell cycle data. The average similarity score with error bars over 100 runs. The estimated underlying component number is three.

Firstly, to prevent the over-learning problem the dimension of the data set was reduced using the input sample selection procedure described in the Methods section. We used the mutual information quality index, *f* (*i,k*−*1*) as in Eq. (1), to evaluate the samples for the most suitable number of inputs. Figure 3 shows the sum of mutual information measured for all the input samples in the positive part of the data. As we can see, there is an apparent increase at the dimension of 66. Therefore, we selected 65 samples for the positive part and 62 for the negative part (the figure is not shown here) for further nICA analysis. Secondly, we used the stability-based dimension estimation method to estimate the number of independent components. The results of stability analysis are shown in Figure 4, and an apparent peak is obtained from the averaged pairwise mutual information when the number of components is equal to 3. Thirdly, we applied the nICA learning algorithm to uncover the independent components for gene module identification. Finally, we obtained the gene modules by gene clustering using VISDA in the latent space. The four most significant gene clusters are given in Table 1. We measured the biological significance of each cluster using the BiNGO tool. The p-value of each cluster was calculated according to its overlap with the functional annotations in Gene Ontology (GO) (see (Maere et al. 2005) for the detail).

### Yeast dataset

Yeast dataset, which exhibits highly coordinated metabolic fluctuations, gene expression patterns and cell division cycles, was cultured under diverse experimental conditions, temperature shocks, amino acid starvation, and progression into stationary phase (Gasch et al. 2000). This dataset has been extensively studied because of its importance in a variety of biotechnological applications. As in (Lee and Batzoglou, 2003), we also used KNNimpute to fill in the missing values (Troyanskaya et al. 2001). And due to the triviality of clustering environmental stress response (ESR) genes defined by (Gasch et al. 2000), we eliminated them in our analysis. The final data set contains 5,284 genes and 173 samples.

**Figure 5:**
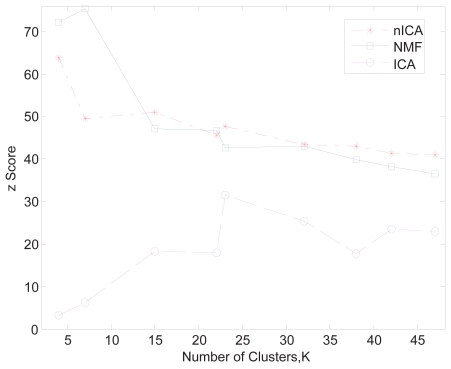
Comparison of clustering results obtained by nICA (asterisk), NMF (square)and ICA (circle), respectively. The data set used for comparison is Dataset 2 of yeast in various stressful conditions (Gasch et al. 2000).

**Figure 6:**
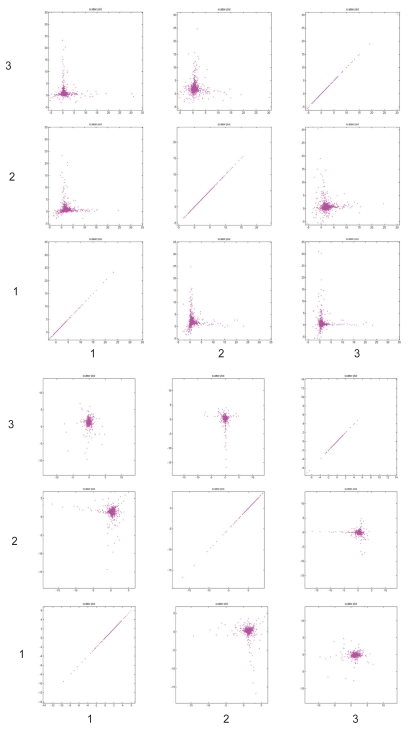
Scatter plots of the first three independent components from nICA (upper) and ICA (lower), respectively. Each sub-panel shows two subsequent components plotted against each other. Evidently, the process-specific genes from nICA are highly biased onto two nonnegative axes (upper), whereas the results from ICA are not (lower).

In this case study, we focus on comparing the result from the nICA approach, which is enforced by the non-negativity constraint, with that from conventional ICA approach (Lee and Batzoglou, 2003) and NMF (another non-negative matrix decomposition approach) (Kim and Tidor, 2003; Lee and Seung, 1999). To objectively evaluate the clustering results from different methods, we used the z-score introduced in (Gibbons and Roth, 2002) to conduct a comparative study. As described in (Gibbons and Roth, 2002), the z-core is based on the mutual information between clustering results and the gene annotation. The higher scores indicate clustering results more biologically significant. We compared the clustering results of nICA, NMF and ICA from small to larger cluster numbers and the z-scores are shown in Figure 5. As we can see from Figure 5, nICA consistently outperformed ICA with an average increase of z-score of 10. It is interesting to observe that the NMF performed slightly better that nICA when the number of cluster is small and nICA performed slightly better than NMF when the number of cluster becomes large. In our opinion, the overall performances of nICA and NMF are comparable. Figure 6 shows the scatter plots of the first three independent components from nICA and ICA, respectively. Since the process-specific genes are highly biased onto two orthogonal axes respectively shown in each sub-panel of Figure 6, we conclude that, comparing with ICA, nICA is quite effective in extracting non-negative independent biological processes. Finally, in Table 2, we list five of the identified co-regulated gene groups that show significant enrichment in GO term categories.

### Muscle regeneration data

We further applied the nICA approach to a time course microarray data set from a profiling study of *in vivo* murine muscle regeneration. Staged skeletal muscle degeneration/regeneration was induced by injection of cardiotoxin (CTX) as described (Zhao et al. 2003). Mice were injected in gastrocnemius muscles of both sides, and then sacrificed at the following 27 time points: 0h(our), 12h, 1d(ay), 2d, 3d, 3.5d, 4d, 4.5d, 5d, 5.5d, 6d, 6.5d, 7d, 7.5d, 8d, 8.5d, 9d, 9.5d, 10d, 11d, 12d, 13d, 14d, 16d, 20d, 30d, and 40d (Zhao et al. 2003). Expression profiles were obtained with Affymetrix’s U74Av2 and MAS 5.0 summarization algorithm. As a preprocessing step, we used the last time point as the reference point and the expression matrix consists of log-ratios of the expression measurements with respect to the reference point.

**Figure 7:**
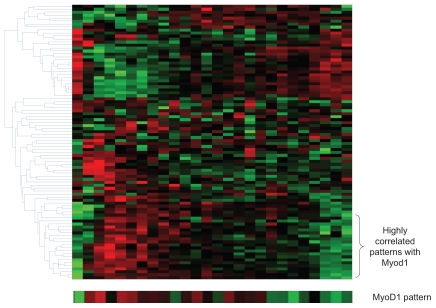
The heatmap of the cluster 8 from the positive part of muscle regeneration data, showing a highly correlated expression pattern with MyoD1 gene.

We then applied the nICA approach to the positive and negative parts respectively for gene module identification. As a result, we found 11 clusters from the positive part of the data and 9 clusters from the negative part; all with significant biological coherence. Several clusters showed an expression pattern highly correlated with *MyoD1* gene (Figure 7 shows an example of the heatmap of cluster 8 from the positive part of the data). *MyoD1* has been widely studied for its important function in embryonic myogenesis and postnatal muscle regeneration. We also examined the biological relevance of these clusters. The results are shown in Table 3 and Table 4 (p-value less than 10^−4^ is considered as significant).

**Figure 8:**
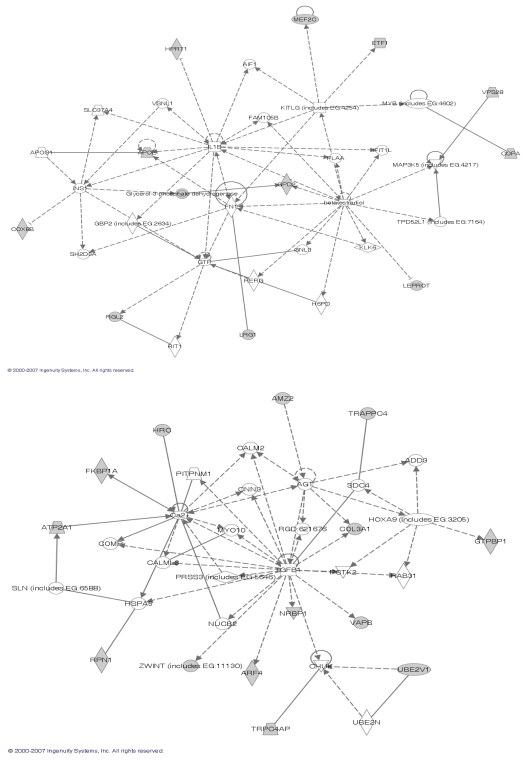
The top panel shows the cluster 6 found in the positive part of the muscle regeneration data with the following functions: post-translational modification, cellular growth and proliferation, and skeletal and muscular system development; The bottom panel shows the cluster 6 found in the negative part of the data with the main function of skeletal and muscular system development.

With the identified gene clusters, we further used the Ingenuity Pathway Analysis (IPA) [10] to assess their biological plausibility, with respect to known information about gene regulatory networks, pathways and module functions. Among them, we found two clusters whose network functions are tightly related to skeletal and muscular system development (Fig. 8). Moreover, the cluster 13 found in the negative part contains *Rb1* and cluster 1 found in the negative part contains *MyoD1*, which are two novel downstream targets of *MyoD* (Bakay et al. 2006).

## Conclusion

This paper presents a new gene clustering approach, namely nICA-based approach, for composite gene module discovery. A complete algorithm of the nICA approach has been developed with the following main components: (1) input sample selection, (2) stability-based dimension estimation, (3) nICA learning algorithm and (4) gene clustering by VISDA. By projecting the gene expression data onto nICA space, co-regulation structures of the modules can be revealed and highlighted. Using a pre-screening and VISDA clustering procedure, we can identify biological process enriched clusters with coherent functional annotations. The experimental results on the yeast data sets have demonstrated the advantages of the nICA approach over conventional ICA-based approach. The results also indicated that the performances of nICA and NMF are comparable. Further, the nICA approach has been applied to a muscle regeneration data set for novel gene module discovery. The results have shown that not only the identified gene modules are biologically significant and plausible, but novel downstream target genes can also be discovered by the nICA approach.

## Figures and Tables

**Table 1:** The four most significant clusters from nICA for the cell cycle data set. Numbers in parentheses in the fifth column show the percentage of genes within the cluster that are presented in one of the functional category. And the numbers in the sixth column are presented in the similar way which corresponds to the total number within the whole genome set that are annotated with one of the special categories in GO system.

Cluster ID	GOID	GO term	p-value	cluster frequency	total frequency
1	6365	35S primary transcript processing	1.27E-27	26/102 (25.4%)	76/5638 (1.3%)
	42255	ribosome assembly	8.87E-18	18/102 (17.6%)	62/5638 (1.0%)
	42273	ribosomal large subunit biogenesis and assembly	1.00E-17	13/102 (12.7%)	23/5638 (0.4%)
	30490	processing of 20S pre-rRNA	2.53E-16	15/102 (14.7%)	43/5638 (0.7%)
	30489	processing of 27S pre-rRNA	8.10E-10	7/102 (6.8%)	13/5638 (0.2%)
2	6511	ubiquitin-dependent protein catabolic process	7.43E-06	11/86 (12.7%)	140/5638 (2.4%)
	19941	modification-dependent protein catabolic process	7.43E-06	11/86 (12.7%)	140/5638 (2.4%)
	51603	proteolysis involved in cellular protein catabolic process	8.52E-06	11/86 (12.7%)	142/5638 (2.5%)
7	7017	microtubule-based process	1.38E-07	13/126 (10.3%)	95/5638 (1.6%)
	7067	mitosis	2.85E-06	13/126 (10.3%)	123/5638 (2.1%)
	16359	mitotic sister chromatid segregation	3.26E-06	9/126 (7.1%)	56/5638 (0.9%)
	7010	cytoskeleton organization and biogenesis	5.30E-06	17/126 (13.4%)	217/5638 (3.8%)
	7059	chromosome segregation	6.09E-06	12/126 (9.5%)	112/5638 (1.9%)
13	19941	modification-dependent protein catabolic process	2.69E-06	10/63 (15.8%)	140/5638 (2.4%)
	51603	proteolysis involved in cellular protein catabolic process	3.06E-06	10/63 (15.8%)	142/5638 (2.5%)
	43632	modification-dependent macromolecule catabolic process	4.18E-06	10/63 (15.8%)	147/5638 (2.6%)

**Table 2:** The five most significant clusters from nICA for the yeast data set.

Cluster ID	GOID	GO term	p-value	cluster frequency	total frequency
13	4386	helicase activity	1.10E-10	11/47 (23.4%)	82/7288 (1.1%)
15	19752	carboxylic acid metabolic process	4.83E-15	17/28 (60.7%)	308/7288 (4.2%)
	6519	amino acid and derivative metabolic process	7.39E-15	15/28 (53.6%)	200/7288 (2.7%)
	6807	nitrogen compound metabolic process	1.49E-13	15/28 (53.6%)	244/7288 (3.3%)
	6144	purine base metabolic process	7.41E-12	7/28 (25.0%)	16/7288 (0.2%)
	103	sulfate assimilation	4.56E-11	6/28 (21.4%)	10/7288 (0.1%)
	6555	methionine metabolic process	1.56E-10	7/28 (25.0%)	23/7288 (0.3%)
16	6807	nitrogen compound metabolic process	1.04E-17	24/62 (38.7%)	244/7288 (3.3%)
	6519	amino acid and derivative metabolic process	1.99E-14	20/62 (32.3%)	200/7288 (2.7%)
18	32197	transposition, RNA-mediated	7.19E-11	13/57 (22.8%)	95/7288 (1.3%)
	3964	RNA-directed DNA polymerase activity	5.25E-10	10/57 (17.5%)	52/7288 (0.7%)
22	6091	generation of precursor metabolites and energy	2.34E-20	25/44 (56.8%)	336/7288 (4.6%)
	6119	oxidative phosphorylation	4.02E-17	13/44 (29.5%)	46/7288 (0.6%)
	6732	coenzyme metabolic process	1.55E-13	15/44 (34.1%)	135/7288 (1.9%)
	42775	organelle ATP synthesis coupled electron transport	1.91E-12	9/44 (20.5%)	25/7288 (0.3%)
	51186	cofactor metabolic process	4.28E-12	15/44 (34.1%)	168/7288 (2.3%)
	15980	energy derivation by oxidation of organic compounds	5.46E-12	18/44 (40.9%)	298/7288 (4.1%)
	6084	acetyl-CoA metabolic process	2.43E-11	8/44 (18.2%)	20/7288 (0.3%)
	9060	aerobic respiration	1.77E-10	11/44 (25.0%)	80/7288 (1.1%)

**Table 3:** The five significant clusters from nICA for the muscle regeneration data set (Positive Part).

Cluster ID	GOID	GO term	p-value	cluster frequency	total frequency
2	7156	homophilic cell adhesion	1.56E-16	22/235 (9.3%)	142/15873 (0.8%)
	16337	cell-cell adhesion	7.87E-12	22/235 (9.3%)	238/15873 (1.4%)
6	7399	nervous system development	1.34E-04	7/29 (24.1%)	651/15873 (4.1%)
	30900	forebrain development	2.12E-04	3/29 (10.3%)	64/15873 (0.4%)
	48731	system development	2.23E-04	7/29 (24.1%)	707/15873 (4.4%)
	48856	anatomical structure development	4.11E-04	11/29 (37.9%)	1962/15873 (12.3%)
8	6886	intracellular protein transport	1.19E-04	10/81 (12.3%)	463/15873 (2.9%)
	6091	generation of precursor metabolites and energy	1.82E-04	12/81 (14.8%)	688/15873 (4.3%)
11	6334	nucleosome assembly	7.26E-14	11/49 (22.4%)	121/15873 (0.7%)
	31497	chromatin assembly	2.68E-13	11/49 (22.4%)	136/15873 (0.8%)
	6325	establishment and/or maintenance of chromatin architecture	2.06E-09	11/49 (22.4%)	311/15873 (1.9%)
	6461	protein complex assembly	2.43E-09	11/49 (22.4%)	316/15873 (1.9%)
	6323	DNA packaging	2.77E-09	11/49 (22.4%)	320/15873 (2.0%)
	7001	chromosome organization and biogenesis (sensu Eukaryota)	3.10E-08	11/49 (22.4%)	404/15873 (2.5%)
15	6092	main pathway of carbohydrate metabolism	4.99E-10	13/170 (7.6%)	120/15873 (0.7%)
	6096	glycolysis	2.59E-09	10/170 (5.8%)	68/15873 (0.4%)
	15980	energy derivation by oxidation of organic compounds	4.66E-09	14/170 (8.2%)	172/15873 (1.0%)
	44265	cellular macromolecule catabolic process	1.42E-08	18/170 (10.5%)	327/15873 (2.0%)
	46365	monosaccharide catabolic process	1.49E-08	10/170 (5.8%)	81/15873 (0.5%)
	19320	hexose catabolic process	1.49E-08	10/170 (5.8%)	81/15873 (0.5%)
	46164	alcohol catabolic process	1.89E-08	10/170 (5.8%)	83/15873 (0.5%)

**Table 4:** The nine significant clusters from nICA for the muscle regeneration data set (negative part).

Cluster ID	GOID	GO term	p-value	cluster frequency	total frequency
1	44238	primary metabolic process	3.29E-05	82/128 (64.0%)	7330/15873 (46.1%)
	19538	protein metabolic process	5.11E-05	48/128 (37.5%)	3504/15873 (22.0%)
2	6096	glycolysis	3.99E-06	7/153 (4.5%)	68/15873 (0.4%)
	6007	glucose catabolic process	8.44E-06	7/153 (4.5%)	76/15873 (0.4%)
3	51641	cellular localization	3.69E-07	23/141 (16.3%)	780/15873 (4.9%)
	46907	intracellular transport	2.93E-06	21/141 (14.8%)	751/15873 (4.7%)
4	7156	homophilic cell adhesion	1.71E-20	22/155 (14.1%)	142/15873 (0.8%)
	16337	cell-cell adhesion	1.38E-15	22/155 (14.1%)	238/15873 (1.4%)
6	15992	proton transport	4.78E-05	5/83 (6.0%)	76/15873 (0.4%)
	6818	hydrogen transport	6.89E-05	5/83 (6.0%)	82/15873 (0.5%)
8	16043	cellular component organization and biogenesis	2.67E-11	63/199 (31.6%)	2146/15873 (13.5%)
	46907	intracellular transport	2.72E-10	33/199 (16.5%)	751/15873 (4.7%)
	51649	establishment of cellular localization	4.37E-10	33/199 (16.5%)	765/15873 (4.8%)
	6996	organelle organization and biogenesis	7.31E-07	36/199 (18.0%)	1197/15873 (7.5%)
	6886	intracellular protein transport	1.55E-06	20/199 (10.0%)	463/15873 (2.9%)
9	6457	protein folding	6.63E-07	19/331 (5.7%)	239/15873 (1.5%)
12	44260	cellular macromolecule metabolic process	2.48E-06	62/174 (35.6%)	3256/15873 (20.5%)
	44267	cellular protein metabolic process	7.48E-06	60/174 (34.4%)	3212/15873 (20.2%)
13	31497	chromatin assembly	2.43E-13	16/153 (10.4%)	136/15873 (0.8%)
	6334	nucleosome assembly	1.02E-11	14/153 (9.1%)	121/15873 (0.7%)
	6333	chromatin assembly or disassembly	1.23E-11	16/153 (10.4%)	175/15873 (1.1%)
